# Extending Delivery of Seasonal Malaria Chemoprevention to Children Aged 5–10 Years in Chad: A Mixed-Methods Study

**DOI:** 10.9745/GHSP-D-21-00161

**Published:** 2022-02-28

**Authors:** Azoukalné Moukénet, Laura Donovan, Beakgoubé Honoré, Kevin Baker, Helen Smith, Sol Richardson, Charlotte Ward

**Affiliations:** a Malaria Consortium Chad Country Office, N’Djamena, Chad.; b Malaria Consortium, London, United Kingdom.; c Independent consultant, International Health Consulting Services Ltd., United Kingdom.; d London School of Hygiene and Tropical Medicine, United Kingdom.

## Abstract

**Background:**

To prevent malaria among children aged 3–59 months in areas with high seasonal transmission, seasonal malaria chemoprevention (SMC) is recommended. In Chad, there is evidence of SMC administration to children aged older than 5 years (referred to as “leakage”). This study aimed to understand the reasons for leakage and explore the feasibility and acceptability of extending the delivery of SMC to children aged 5–10 years in Chad.

**Methods:**

We conducted a mixed-methods study in Massaguet health district with a cross-sectional survey to determine SMC coverage for children aged up to 10 years after SMC cycles 1 and 3 (n=90 and n=100 caregivers surveyed, respectively) and at the end of cycle 4 (n=101 caregivers surveyed). We conducted 14 key informant interviews at the national and district level and 8 focus group discussions with community distributors and caregivers.

**Results:**

In the compounds surveyed, there were no children aged 5–10 years in cycle 1. In cycles 3 (n=1 children) and 4 (n=16 children), there was 100% (95% confidence interval [CI]=2.5, 100.0) and 62.5% (95% CI=35.4, 84.8) coverage of SMC in children aged 5–10 years, respectively. Extension of SMC to older children was considered acceptable, but there were concerns about feasibility and ensuring the sustainability of the current program in children aged 3–59 months. Key informants acknowledged the need to secure additional funding to pilot SMC in older age groups and were uncertain about the impact of the current SMC program at scale.

**Conclusion:**

Key informants considered extending SMC to children aged 5–10 years acceptable but did not deem it a current priority. They expressed an urgent need to address leakage and reinforce both the sustainability and quality of the current SMC program.

## Abstract (precis)

We sought to understand perceptions of the feasibility and acceptability of extending seasonal malaria chemoprevention (SMC) to children aged 5–10 years and explore reasons why SMC is administered to children aged 5–10 years in the current program.

## Teaser

**Key Findings:**

- Extending the age eligibility for seasonal malaria chemoprevention to children aged 5–10 years is generally regarded as acceptable by key informants in Massaguet district, Chad.
- Decision makers at the policy level consider it more urgent to achieve full coverage, demonstrate impact, and ensure the sustainability of SMC administration in children aged 3–59 months in the current program.

**Key Implications:**

Leakage in the current program, resulting in children aged older than 59 months receiving SMC, is commonly acknowledged at all levels of the health system. To address this, program managers should consider more stringent selection criteria for community distributors, as well as more frequent training and more extensive sensitization at the community level.

Résumé en français à la fin de l'article.

See related article by Hodgins.

## BACKGROUND

In sub-Saharan Africa, malaria is a major public health problem.^1^^–^^3^ To reduce the burden of malaria among children, the World Health Organization recommends the use of sulfadoxine-pyrimethamine (SP) and amodiaquine (AQ) to prevent malaria in children aged 3–59 months in areas with high seasonal transmission of malaria.^4^^,^^5^ In most settings in the Sahel, seasonal malaria chemoprevention (SMC) is implemented door-to-door by volunteer community distributors^6^ during the high transmission season.

Malaria is endemic in Chad, with a prevalence of 40.9% in children aged younger than 5 years and 41.3% in children aged 5–14 years at the end of the rainy season (October–November) in 2017.^7^ SMC was first introduced in 2013 and scaled up from 2014. Since 2015, Malaria Consortium has provided operational support in 14 districts, including drug procurement, training, supervision, monitoring, and evaluation. In 2019, SMC was implemented in 41 (67.2%) eligible districts in Chad; Malaria Consortium supported 20 of these districts. Health facilities are supplied with drugs through the national logistics chain, and SMC is delivered by community distributors who are supervised by health center managers. The program is under the supervision of the Provincial Health Delegation and the National Malaria Control Program (NMCP) with support from the Department of Pharmacy, Drugs, and Pharmacopoeia. The first daily dose of SP and AQ is usually administered by a community distributor and the second and third daily doses of AQ are left with the caregiver to administer.^6^ Before each campaign, community distributors are trained on contraindications of the drugs, how to administer the drugs, and how to complete monitoring records as described elsewhere.^4^^,^^6^

Although SMC is recommended for children aged younger than 5 years, there is evidence of “leakage” to children older than 5 years.^8^^–^^10^ This is a challenge because the formulation in the dose for children aged 3–59 months children may not provide full protection for older children during peak malaria transmission; thus contributing to the development of drug-resistant *Plasmodium falciparum* parasites.^9^^,^^11^^,^^12^ The SMC coverage surveys from 2017–2018 suggest that SMC is often administered to children aged older than 5 years with coverage varying between 60–100% in Massaguet district and 30%–64% in all districts implementing SMC.^10^^,^^13^^,^^14^ Reports from monitoring surveys in Chad^15^^,^^16^ suggest that community distributors may be pressured by caregivers to administer SP and AQ to older children and may not adhere to direct observation of first dose administration, creating an opportunity for caregivers to administer SMC to older children.^17^ Studies elsewhere have found that caregivers find it difficult to prioritize SMC for children who are not ill and may reserve the drugs to use for older children who fall ill.^18^ As well as the problems with leakage of SMC doses, in Chad, the use of other malaria control interventions such as long-lasting insecticidal nets is low^7^ among children aged younger than 5 years (52.3%) and children aged 5–14 years (48%), highlighting the importance of increasing long-lasting insecticidal nets coverage or implementing additional interventions.

Furthermore, for the whole country, malaria incidence is higher among children aged 5–14 years than children aged under 5 years, with a peak during the rainy season (July–October).^19^ There is also evidence from Mali that the peak of clinical episodes has shifted from children aged younger than 5 years to older children and extending SMC to children older than 5 years has been suggested.^20^ Based on surveillance data showing the burden of malaria in older children, trials conducted in Senegal indicate extending SMC to children aged younger than 10 years is effective, acceptable, and could contribute to reducing malaria transmission and thus have a greater impact on malaria burden.^21^^,^^22^ In addition, recent high-level technical consultations have discussed opportunities for optimizing SMC including age group expansion.^23^

Given the problems in reaching the target group in the existing SMC program, and the shifting incidence and morbidity trends seen in Chad, there is a need to explore perspectives on extending the delivery of SMC to older children. We sought to understand perceptions of the feasibility and acceptability of extending SMC to children aged 5–10 years and explore reasons for leakage in the delivery of the current program.

Given the problems in reaching children younger than 5 years old in the existing SMC program, there is a need to explore perspectives on extending the delivery of SMC to older children.

## METHODS

### Study Design

We conducted a mixed-methods study using focus group discussions (FGDs), key informant interviews (KIIs), and cross-sectional surveys of SMC coverage.

### Study Setting

This study was conducted in Massaguet health district in Hadjer Lamis province, where SMC has been implemented since 2015. There are 16 health centers that serve a total population of 143,952, including 44,192 children aged 5–10 years and 28,682 children aged younger than 5 years.^24^ Most households in the district are located in rural areas (86.9%),^25^ with high levels of population movement due to trade activities with N’Djamena, farmers commuting from N’Djamena, and nomadic populations’ transhumance. We chose Massaguet as the study site due to operational feasibility and reports of high levels of SMC leakage to children aged 5–10 years in 2018.^14^

This study was developed and overseen by employees of Malaria Consortium; authors based in N’djamena are involved in routine program monitoring and evaluation activities and those in the United Kingdom are responsible for supporting operational research to improve program performance and impact.

### Data Collection

#### Cross-Sectional Survey

Under usual program circumstances, an external research company^26^ routinely conducts a cross-sectional survey at the end of cycles 1 and 3, using the lot quality assurance sampling (LQAS) method as recommended by the WHO.^27^ After the whole campaign, another company conducts an end-of-round coverage survey using a representative sample.^26^ We describe the sampling methodology for these surveys.

#### End of Cycles Survey (LQAS Methodology)

Massaguet district has 16 health centers, subdivided into 5 supervision areas. Within each supervision area, a multistage cluster random sampling technique was used to select 19 compounds. As the first-stage unit, 9 villages were selected using the random function of Excel from the list of villages within the supervision area. At the second-stage unit, about 30 compounds were selected using a lottery method within each village selected. At the third-stage unit, 2 compounds (3 compounds for the last group of compounds) were selected using a lottery method. The sample size of 19 compounds was selected to satisfy the classification requirement of the LQAS: if 13 of these 19 compounds had been visited by SMC community distributors, then the supervision area was classified as having reached the target of 80% coverage. The eligibility criteria for compounds were accepting the survey and having at least 1 child aged under 5 years. The total sample size in Massaguet district is 95 compounds.

#### Coverage Survey

To select the representative sample size for the coverage survey, we used a multistage cluster random sampling technique with the health center as the first-stage unit, villages as the second, and compounds as the third stage. From the 16 health centers, 5 health centers (clusters) including 1 urban health center were selected using the random function of Excel. In each selected health center, 5 villages were randomly selected using the random function of Excel. About 30 compounds were selected using a lottery method within each village selected. Four compounds were selected from each study village in the respective health centers using a lottery method. A total of 100 compounds were selected as the sample size for the survey. The sampling plan for the coverage survey adopted is to provide a more accurate picture of the achievement of SMC objectives and to understand the degree of compliance with the SMC protocol. Sample size calculation was based on SMC coverage of 90% (SMC coverage of 2018 at all rural districts level), a precision of 3% and 90% power. The eligibility criteria were accepting the survey and having at least 1 child under 5 years.

Both surveys use protocols developed by Malaria Consortium, and data were collected at the compound level^26^ using electronic questionnaires on a mobile data collection (Magpi version 6.2.1).^28^ For this study, data from both the end-of-cycle and end-of-round surveys were extracted for Massaguet district and analyzed.

### Qualitative Data Collection

Separate topic guides were designed for community distributors, female caregivers, and key informants using existing frameworks that outline key elements to explore the perceived feasibility,^29^ acceptability,^30^ and implementation^31^ of SMC to children aged 5–10 years. Topic guides were designed with broad open questions and prompts to explore the reasons for SMC leakage to children aged 5–10 years and acceptability, implementation, practicability, integration, and expansion of SMC to older children.

FGDs were held with community distributors and caregivers from 1 rural village and 1 urban settlement who had participated in the 2019 SMC campaign. We held 2 FGDs with female community distributors and 2 with male community distributors per area. We held 4 FGDs with female caregivers (aged older than 30 years and younger than 30 years) of children aged younger than 10 years.

Fourteen KIIs were held in N’Djamena and Massaguet with religious and community leaders, health facility and district health personnel, the NMCP, and the National Drug Authority. Interviewees were purposefully selected based on their position within the SMC program and their availability to participate. Community distributor supervisors and village leaders were purposefully selected based on convenience and availability.

Interviews and FGDs were conducted by independent research assistants, who assured participants of their impartiality to the SMC program and its delivery. Interviews and FGDs were conducted in local Arabic and French.

### Data Analysis

Survey data were analyzed using STATA 16 to process descriptive statistics. Coverage was defined as the proportion of children of a specific age group reported by caregivers as having received the first SMC dose during a cycle among the total number of children reached during surveys. For comparison, we calculated coverage both at the Massaguet district level and at the level of all districts where Malaria Consortium implements SMC. Data used to calculate coverage at all district levels were obtained from the same surveys as those of Massaguet.

Qualitative data were transcribed verbatim from Arabic to French and then translated into English. A sample of transcripts in French and English were checked for transcribing and translation accuracy by 1 author (AM) fluent in Chadian Arabic, French, and English. Data were analyzed thematically^32^ and used MAXQDA^33^ to manage data coding, searching, and retrieval. LD developed initial coding frames for FGDs and interviews, which were discussed with all authors, modified, and then used to code all transcripts. LD collated coded data into categories and then emerging themes. Summaries of each theme were reviewed and discussed by all authors before final consolidation.

### Informed Consent and Ethical Considerations

All study participants were fully informed of the study and had time to consider their participation and ask questions before giving written consent. The study was approved by the National BioEthics Committee of Chad (N°0173/PR/MESRI/SG/CNBT/2020) and the Liverpool School of Tropical Medicine Research Ethics Committee (Ref: 19-071).

## RESULTS

### Routine Data on SMC Coverage

A total of 90 (cycle 1) and 100 (cycle 3) caregivers of children aged 3–59 months participated in the routine LQAS monitoring surveys in Massaguet district. For operational reasons, no survey was implemented in cycle 2. A total of 101 (cycle 4) caregivers participated in the routine end-of-round survey at Massaguet district.

In compounds surveyed during the 2019 SMC cycle 1, there were no children aged 5–10 years. One child aged older than 5 years in Massaguet received at least 1 SMC dose in cycle 3. By cycle 4, 10 children of 16 who were aged older than 5 years in the surveyed compounds received SMC (62.5%) in Massaguet district, which is similar to the coverage in children aged older than 5 years in 20 health districts (Table 1).

**TABLE 1.** Coverage of SMC by Age Group and Cycle in the Health District of Massaguet and in 20 Health Districts Supported by Malaria Consortium, 2019

Age, months	Location	Cycle 1			Cycle 3			Cycle 4		
		n/N	%	95% CI	n/N	%	95% CI	n/N	%	95% CI
3–11	Massaguet	6/6	100.0	54.1, 100.0	79/80	98.8	93.2, 100.0	10/12	83.3	51.6, 97.9
	20 health districts	387/446	86.8	83.3, 89.8	625/731	85.5	82.7, 88.0	455/508	89.6	86.6, 92.1
12–59	Massaguet	260/261	99.6	97.9, 100.0	200/201	99.5	97.3, 100.0	163/167	97.6	94.0, 99.3
	20 health districts	3,764/4,110	91.6	90.7, 92.4	3,570/4,056	88.0	87.0, 89.0	4,758/5,055	94.1	93.4, 94.8
60–108	Massaguet	NA	NA	NA	1/1	100.0	2.5, 100.0	10/16	62.5	35.4, 84.8
	20 health districts	217/511	42.5	38.1, 46.9	85/696	12.2	9.9, 14.9	509/784	64.9	61.5, 68.3

Abbreviations: CI, confidence interval, NA, no data available.

### Qualitative Findings

We conducted 14 KIIs with policy makers and a donor representative at the national level; provincial, district, and zonal managers, as well as the malaria focal point at the district level; health center managers at the facility level; and supervisors of community distributors and village leaders in the community (Table 2).

**TABLE 2.** Roles of Key Informants Interviewed on SMC Coverage, Chad

**Level**	**Organization**	**Role in SMC Program**
National (n=4)	NMCP	Lead the national technical committee of SMC
		Provide technical coordination of SMC activities from planning to implementation and evaluation
	Central Pharmaceutical Drug	Ensure safe storage and quality control of SMC drug
	UNICEF	Support SMC implementation including planning, monitoring and evaluation, activities implementation, and drug supply in relation to national strategy
	National High Consultative Councils	Mobilize resources from the Global Fund and provide strategy orientation
		Conduct monitoring and evaluation of activities implemented by partners including United Nations Development Programme (the main recipient implementing SMC through NMCP)
District (n=4)	Provincial Health Delegation of Hadjer Lamis	Coordinate all health activities and programs in the province including SMC (representing the Ministry of Health)
	District of Massaguet	Conduct activities from planning to implementation and reporting of SMC activities and ensure the SMC implementation in the health centers
		Conduct social mobilization
		Ensure the safe drug storage and dispatching into health centers
Facility (n=2)	Health center	Conduct the SMC implementation, training of community distributors and supervisors, supervision of SMC drug administering, daily data reporting on SMC implementation, and stock reconciliation of drug
Community (n=4)	Community	Supervisors oversee community distributors’ activities including drug administering, data reporting, reference cases, and stock reconciliation
		Village leaders sensitize community to adhere to SMC intervention and solve refusal cases

Abbreviations: NMCP, National Malaria Control Program; SMC, seasonal chemoprevention program.

We conducted 4 FGDs with community distributors and 4 FGDs with caregivers. Sociodemographic characteristics are summarized in Table 3.

**TABLE 3.** Sociodemographic Characteristics of FGD Participants: Community Distributors and Caregivers

	Community Distributors				Caregivers			
	Male		Female		Aged Younger Than 30 Years		Aged Older Than 30 Years	
	Massaguet Urbain (N=8)	N'Djamena Fara (N=8)	Massaguet Urbain (N=8)	N'Djamena Fara (N=7)	Massaguet Urbain (N=8)	N'Djamena Fara (N=8)	Massaguet Urbain (N=8)	N'Djamena Fara (N=8)
Age, years								
18–30	6	7	7	5	8	8	0	0
31–40	2	1	1	2	0	0	6	4
41–50	0	0	0	0	0	0	2	4
Education								
None	0	0	0	1	2	0	1	1
Koranic	0	0	0	0	0	0	0	3
Primary	1	4	1	1	1	0	5	3
Secondary	7	3	7	4	5	8	1	1
Tertiary	0	1	0	1	0	0	1	0
Employment								
Student	5	0	0	0	1	3	0	0
Farmer	1	4	0	0	1	0	1	6
Housewife	0	0	4	2	2	0	1	1
Tailor	0	0	1	0	0	0	0	0
Teacher	0	1	0	0	0	0	0	0
Trader	1	3	3	3	4	5	6	1
Other	1	0	0	2	0	0	0	0
No. years' experience conducting SMC								
1	0	7	3	1	N/A	N/A	N/A	N/A
2	0	0	0	0	N/A	N/A	N/A	N/A
3	0	0	0	0	N/A	N/A	N/A	N/A
4	0	0	3	3	N/A	N/A	N/A	N/A
5	3	1	1	1	N/A	N/A	N/A	N/A
6	5	0	1	1	N/A	N/A	N/A	N/A
Age of children treated, months								
< 3	N/A	N/A	N/A	N/A	0	0	0	0
3–11	N/A	N/A	N/A	N/A	0	0	0	0
12–59	N/A	N/A	N/A	N/A	7	5	6	7
> 60	N/A	N/A	N/A	N/A	1	2	2	1

Abbreviations: N/A, not applicable; SMC, seasonal chemoprevention.

### Acceptability of Extending SMC to Older Children

All key informants supported the extension of SMC because they felt that older children who also require protection from malaria should receive SMC. Some key informants also felt that the extension would ease the case management workload in health facilities.

*Our role would become very easy now because our structures do not have an adequate technical platform to take care in a curative way of malaria … and it will relieve our health facilities than letting children die for avoidable deaths.* —KII, Provincial Health Delegate, Hadjer-Lamis

All key informants supported the extension of SMC because they felt that older children who also require protection from malaria should receive SMC.

Others felt that extending SMC would ease community distributors’ workload because older children could act as role models, encouraging SMC adherence or administering the drug to their younger siblings in the absence of the caregiver.

*When the big one swallows, he will give to his little brothers even if his father and mother are absent. If the community distributor explains it very well, he will give all his little ones. There will be no problem*. —KII, Zone manager

Caregivers also mentioned that giving SMC to older children would reduce the burden of malaria. They anticipated fewer episodes of malaria and less expenditure on treatment, allowing them to use available income to send children to school. They also held the view that if older children were protected from malaria, they would be able to help in the fields with farming activities. Some even requested Malaria Consortium to extend SMC to the adult population too.

*This will allow us to rest from malaria and spend less money as a result of malaria. During the rainy season, if you have some money, you will eat and have the strength to plough more. Instead of spending everything on care.* —FGD, Female community distributor, Fara

### Feasibility of Extending SMC to Older Children

Key informants seemed concerned about the logistical and financial feasibility of extending SMC to older age groups. Concerns included whether older children would be available during distribution cycles when they are attending school or working in the fields, determining the correct drug dosage for different age groups, and identifying the age of older children. Despite this, many supported the idea and felt that the challenges could be overcome. Many community distributors expressed concern over the increased workload that could accompany an extension of the SMC program. Caregivers were overwhelmingly positive about the suggestion of extending SMC and optimistic about the benefits.

*Already people have the trouble to identify the age of 5-year-old children, when you say 10-years-old now what will be the criterion to identify a 10-year-old child and include it or not include it in the context of Chad* —KII, Person responsible for health and nutrition, UNICEF

*It's the feasibility that worries me. Will the tablets be even more dosed [provide full protection compared to the ones currently provided] than the ones we are currently giving? How are we going to take it or do it?* —KII, Chief medical officer

Key informants seemed concerned about the logistical and financial feasibility of extending SMC to older age groups.

#### Resource and Remuneration Requirements

Key informants were aware that the resources currently allocated to the program were insufficient to implement an extension of SMC to older children. To avoid significantly increasing the workload of community distributors, they recognized that the number of distributors and days of SMC distribution would need to increase. Key informants highlighted the need to provide transport for community distributors working in villages located far from health centers, as well as remuneration for food and fuel, which were often regarded as an incentive for good work. They also noted that sufficient drug supply would need to be procured and made available well before the start of the malaria transmission season.

*Also think of the means of transport for community distributors. The person comes to help you with their logistics. You give him 2,500 or 3,000. Of that amount, he has to pay for fuel, he has to look for food. If he goes into the bush and there is a breakdown … we must first correct these shortcomings. You have to fix everything before expanding*. —KII, Zone manager

*If there is not an increase in all these resources it is not worth expanding the SMC, the current resources in place are not sufficient to make the extension… with the increase, community distributors will spend more time in households. The more there is, the more community distributors are needed.* —KII, Coordinator, NMCP

#### Program and Health System Requirements

Key informants highlighted the need for research evidence on the impact of age group extension on malaria morbidity and mortality. Furthermore, most felt it would be necessary to first achieve full coverage of SMC in children 3–59 months in all eligible districts in Chad. In addition, they highlighted the need for awareness-raising among caregivers before the rainy season with the involvement of key community actors. Key informants were also keen to identify the total cost of extending the program and understand the financial and logistical feasibility and sustainability.

*Before embarking on such arrangements it is necessary to see the feasibility and the sustainability. This is rather what I think we should analyze. Is it doable? Is it sustainable?* —KII, Person responsible for health and nutrition, UNICEF

*The administration will be successful based on the sharing of information, by sensitizing the beneficiaries on the new formula to adopt, it is necessary to bring together the community leaders and give them the task that remains it is up to them to do so.* —KII, Village leader

Key informants highlighted the need for research evidence on the impact of age group extension on malaria morbidity and mortality.

### Reasons for Leakage of SMC Into Older Age Groups

#### Awareness and Impact of Leakage on the Current Program

Key informants at the national level were aware that SMC is often incorrectly administered to children aged older than 5 years and explained it also occurred in other health campaigns. However, some felt that it was unacceptable to administer SMC to older children because the public health effect is not achieved in the target group.

*This is a global problem in Chad, it is not only for SMC. All interventions that affect children, I take for example vaccination, vitamin A supplementation and deworming. It is a known fact in Chad. It is that workers, whether volunteers or health workers, tend to give to people who are not the target.* —KII, Person responsible for health and nutrition, UNICEF

*So the fact of vaccinating [treated] the oldest is at the expense of the least and in general we do not have the target. The desired effect in terms of public health is not achieved by not protecting the most vulnerable.* —KII, Person responsible for health and nutrition, UNICEF

#### Reasons for Leakage

Key informants and community distributors expressed that the primary reason for leakage was pressure from caregivers, who they perceived did not understand the importance of treating only children aged younger than 5 years. Community distributors reported being threatened by caregivers who claimed that if their older children are not treated, they would not allow their other eligible children to be treated in the next SMC cycle. Beliefs about SMC drugs also seemed to drive use in older age groups. For example, some health facility staff believed SMC could prevent malaria in children aged 5 years and older. Caregivers also believed that SMC could cure malaria in adults.

*In some households where we find 5 children among which 2 are over 5 years old for example, the mothers ask us to also administer to the 2 children over 5 years old. Otherwise, we will not be back soon. We are also obliged to administer to the latter who are not eligible.* —FGD, Female community distributor, Fara

*Some people in the field do it without knowing it, but my child is 6 years old and I prefer to give it to him because I think it will cover him against malaria.* —KII, Chief medical officer

*Me, this day, malaria bothered me. I had been taking other medications for a long time but it still didn’t work. And I was told to take the SMC medication, I took it. When I took it, I got healthy.* —FGD, Caregiver, Fara

Community distributors reported being threatened by caregivers who claimed that if their older children are not treated, they would not allow their other eligible children to be treated in the next SMC cycle.

Key informants pointed out the lack of drug stock management including monitoring of supply and allocation at the health center level as a factor for enabling community distributors to administer to older age groups. Secondly, the target population is underestimated as it is obtained from outdated census estimates from 2009, thus supervisors are less likely to ensure that community distributors follow eligibility criteria and community distributors treat additional children, facilitating leakage to older children. In addition, health centers and districts are created each year, making it difficult for the Ministry of Health to have an accurate estimate of the population in areas covered by different districts. Thus, distributors were able to request additional drugs to cover any children not identified in the underestimates. In addition, participants highlighted the difficulty in tracking highly mobile nomadic populations, who were often not included in the enumeration data.

*No! Administration of SMC in those over five years of age does not affect the target. Because before going to households, we take the tablets in good quantity [enough].* —FGD, Male community distributor, Fara

*As I said in fact it happened that each time when we have overshoots of the planned inputs and what is treated among these arguments there is a problem of data by what we have underestimated the population, either during the census since it is during the rainy season that the SMC is made. There must be displacements like for example people on the other side who come to an area where they do their rural work, where there are displacements of the nomadic population and so on.* —KII, Coordinator, NMCP

Community distributors also highlighted difficulties in determining the age of children, which they attributed to illiterate parents, and the difficulty they had in distinguishing between children aged 5 and 6 years. Community distributors explained that estimating a child’s age through observing them put their hand over their head, was not always accurate.

*It is not easy to recognize the age of children. It’s the mom who’s supposed to know. But there are some who do not know the age of their children. When we ask them, they tell us that the child has not yet lost baby teeth. How can we know the age of the child? Sometimes, even at seven or eight years old, the baby does not lose his baby teeth yet.* —FGD, Female community distributor, Fara

*Some requests to put your arm over your head. [Laughs]. Nowadays, the size of children varies. How will you know?* —FGD, Male community distributor, Fara

### Preventing Leakage in Future SMC Rounds

Key informants suggested additional training for community distributors before each SMC cycle since staff turnover is high; the training should focus on reminding them of the age eligibility.

*You have to train the actors in the field to properly determine the ages and also give them good arguments to be able to convince parents who insist that we can give drugs to children off target. So, it is much more the enumeration, a good training of actors in the field and a good awareness of the population* —KII, Provincial health delegate, Hadjer-Lamis

Awareness-raising and sensitization, both before and during the campaign, among caregivers and fathers were recognized as key to explain and reinforce age eligibility for SMC. Some key informants also insisted on the need to involve existing community structures in social mobilization as they are most listened to and respected within the community.

*Mass communication is not only mothers especially in the context of Chad, the mother is important but I think the father is important too. Because in Chad, especially in certain areas, it is not the mother who decides it is the man who decides. So if you want to change behaviours or if you want to have a membership, you have to educate the mother as well but you have to educate the father too… You have to work with existing community structures, with traditional chiefs, customary, administrative and whatever you want because they are more listened to in the community.* —KII, Person responsible for health and nutrition, UNICEF

Awareness-raising and sensitization, both before and during the campaign, among caregivers and fathers were recognized as key to preventing leakage to explain and reinforce age eligibility for SMC.

Other solutions included a more rigorous process to recruit community distributors by following predefined selection criteria; literacy was identified as an important criterion to enable community distributors to identify the age of the children and improve their ability to explain the importance of age eligibility. Being known by the local community was also identified as a criterion for recruitment; since this would increase community distributors’ ability to know the age of the children and build trust with caregivers to treat only eligible children.

*We have a big selection problem here. Selection in many cases I realize that it is not based on skills or prerequisites… If you want a volunteer to be good, you must meet the minimum criteria that you have set. Can the person read? Can she write? Does he come from the community? Because I have cases here where we have taken people who are not even from the community concerned to go and do community activities. A community to which you are foreign there is a barrier already when you come to this community.* —KII, Person responsible for health and nutrition, UNICEF

## DISCUSSION

This is the first study to explore the feasibility and acceptability of SMC extension in Chad and the factors facilitating SMC leakage into older age groups.

Extension of SMC to older children was acceptable to stakeholders in this study, due to the perceived protection it offers against malaria in children 3–59 months old. Protecting older children from malaria through SMC is seen as a way to ease the workload of health facility staff during the rainy season, especially when recent data show an increase in malaria cases and admissions among children up to 14 years.^34^ This is similar to other recent studies conducted in the Sahel.^22^ In addition, with 46.7% of the population of Chad identified as “poor,”^35^ administering SMC to older children is a potential way of reducing the out-of-pocket expenditure for caregivers. There was also a demand for malaria chemoprophylaxis for adults, which could be intensified by extending the age range in children.

Concerns were raised about the feasibility of extending the SMC program to older children without evidence of the impact of the current program in children 3–59 months at scale in reducing the incidence of malaria. Furthermore, as highlighted by the policy makers in this study, extending the age range of the SMC program in Chad will first require evidence of its feasibility and acceptability, which has been reported elsewhere in the region.^36^ Resource requirements for adapting the current program would be significant, and for some, this was not justified when full coverage of SMC in the intended population (3–59 months) had not been achieved.

Resource requirements for adapting the current program would be significant, and for some, this was not justified when full coverage of SMC in the intended population (3–59 months) had not been achieved.

Results highlight concerns about the time and resource implications associated with implementing SMC in older children. Some key informants argued that an adapted program would require increased remuneration for and hiring of additional community distributors. This is contrary to results from a pilot study assessing the feasibility of extending the age range of SMC in Senegal that found that older children could readily be treated during distributor household visits with little impact on the time required.^21^ In addition, a challenge with increasing remuneration is that health center and district managers may take the opportunity to pocket the additional remuneration through fraudulent activity, in a context where program monitoring and governance is poor.^37^

There is a need to address leakage of SMC to older children in the current program, which seems to be common particularly when community distributors carelessly apply the age eligibility criteria,^8^ malaria remains the primary cause of health care seeking in Chad,^7^^,^^38^ and there is increasing demand for malaria prevention strategies. Caregivers believe SMC can prevent malaria for older children, and this has led to increased pressure on community distributors to administer the drugs to older children. Similar findings from Burkina Faso explain the demand for SMC in older children is due to caregiver enthusiasm for the SMC strategy in preventing malaria.^8^

In relation to accidental leakage caused by carelessly applying age criteria, the census data used to identify eligible children are outdated and of poor quality. With the estimated number of eligible children frequently incorrect, there is an opportunity for surplus drugs to be administered to older children. Accurately determining a child’s age is another challenge. Community distributors are trained to identify age through morphology, yet, this method is not always reliable due to the high prevalence of malnutrition and stunting.^39^ This combined with low literacy levels among female caregivers (22.1% of women in Chad^40^), lack of consistent provision of identification documents, and an underdeveloped civil registration and identification system (less than 10% of births registered have been issued a birth certificate^40^), makes it difficult for caregivers to know the accurate age of their children.

Factors facilitating the problem of intentional leakage are various and cannot be solved completely, but they can be minimized. Stakeholders suggested the need to improve the knowledge and skills of community distributors to explain the importance of age eligibility and enable them to resist pressure from caregivers. Currently, community distributors are recruited based on their literacy level and being members of the community. However, in a context where just 43.9% of men and 14.3% of women in rural areas are literate,^41^ and many villages do not have the required number of literate community distributors, pairing a nonliterate community distributor with another from a different community can help.

Stakeholders suggested the need to improve the knowledge and skills of community distributors to explain the importance of age eligibility and enable them to resist pressure from caregivers.

Regarding training, a longitudinal study in Burkina Faso found that capacities of community distributors grew from round to round and campaign to campaign, after most had undergone training and been supervised.^42^ However, in the context of Chad, retaining community distributors is a challenge since as many of them are students; at cycle 3, most of them leave villages for school or become preoccupied with their studies. Therefore, health centers need to reconsider the timing of training. Training also needs to include strategies to help distributors resist pressure from caregivers to administer SMC to older children. Combining training with supervision or using multifaceted strategies including group problem solving may help to improve the quality of SMC provision.^43^

### Limitations

The study did not explore the specific views of highly mobile nomadic populations, whose opinions on age extension of SMC may be different. The monitoring and end of round coverage surveys were powered for children aged 3–59 months old thus the estimated coverage indicators for older children will be less precise.

## CONCLUSION

Extending SMC to children aged 5–10 years in Chad was acceptable to national-, district-, facility-, and community-level stakeholders in 1 district of Chad. However, addressing the challenges in the existing program is considered a higher priority by decision makers at the policy level. They expressed the need to achieve full geographical coverage, demonstrate impact at scale, and ensure sustainability of SMC administration in children aged 3–59 months. In addition, solutions are needed to prevent leakage and wastage of scarce resources, which has the potential to reduce the program’s health impact in the intended age group. Key informants and community distributors offered potential solutions to address pervasive problems at the program and health system levels. Future work could involve the use of end-of-cycle survey data from subsequent years and across other SMC countries, to investigate the “leakage.”

## Translation

**En Français**

**L'extension de L'administration de la Chimioprévention du Paludisme Saisonnier aux enfants Agés de 5 à 10 Ans au Tchad: Une Etude à Méthodes Mixtes**

**Principales conclusions**

L'extension de l'âge d'éligibilité pour la chimioprévention du paludisme saisonnier aux enfants âgés de 5 à 10 ans est généralement considérée comme acceptable par les informateurs clés du district de Massaguet, au Tchad.

Les décideurs au niveau des politiques considèrent qu'il est plus urgent d'atteindre une couverture totale, de démontrer l'impact et d'assurer la durabilité de l'administration de la CPS chez les enfants âgés de 3 à 59 mois dans le programme actuel.

**Principales répercussions**

Les fuites dans le programme actuel, qui font que des enfants âgés de plus de 59 mois bénéficient de la CPS, sont communément reconnues à tous les niveaux du système de santé. Pour y remédier, les responsables du programme devraient envisager des critères de sélection plus stricts pour les distributeurs communautaires, ainsi que des formations plus fréquentes et une sensibilisation plus étendue au niveau communautaire.

**RÉSUMÉ**

**Contexte:** Afin de prévenir le paludisme chez les enfants âgés de 3 à 59 mois dans les zones à forte transmission saisonnière, la chimioprévention du paludisme saisonnier (CPS) est recommandée. Au Tchad, il existe des preuves de l'administration de la CPS à des enfants âgés de plus de cinq ans (appelée " fuite "). Cette étude visait à comprendre les raisons de cette " fuite " et à explorer la faisabilité et l'acceptabilité de l'extension de l'administration de la CPS aux enfants âgés de 5 à 10 ans au Tchad.

**Méthodes:** Nous avons mené une étude à méthodes mixtes dans le district sanitaire de Massaguet avec une enquête transversale pour déterminer la couverture en CPS des enfants âgés de 10 ans au moins après les cycles 1 et 3 de la CPS (n=90 et n=100 pourvoyeuses de soins interrogées, respectivement) et à la fin du cycle 4 (n=101 pourvoyeuses de soins interrogées). Nous avons mené 14 entretiens avec des informateurs clés au niveau national et au niveau du district, et huit discussions de groupe avec des distributeurs communautaires et des pourvoyeuses de soins.

**Résultats:** Dans les maisons étudiées, il n'y avait aucun enfant âgé de 5 à 10 ans dans le cycle 1. Dans les cycles 3 (n=1 enfant) et 4 (n=16 enfants), il y avait une couverture de 100% (intervalle de confiance [IC] de 95% = -2,5, - 100,0) et 62,5% (IC de 95% = -35,4, - 84,8) de la CPS chez les enfants âgés de 5 à 10 ans, respectivement. L'extension de la CPS aux enfants plus âgés a été jugée acceptable, mais la faisabilité et la pérennité du programme actuel chez les enfants âgés de 3 à 59 mois ont suscité des inquiétudes. Les informateurs clés ont reconnu la nécessité d'obtenir des fonds supplémentaires pour piloter la CPS dans les groupes d'âge plus élevés, - et étaient indécis quant à l'impact du programme actuel de CPS à plus grande échelle.

**Conclusion**: Les informateurs clés ont jugé acceptable l'extension de la CPS aux enfants âgés de 5 à 10 ans, mais ne l'ont pas considéré comme une priorité actuelle. Ils ont exprimé un besoin urgent de s'attaquer aux fuites et de renforcer à la fois la durabilité et la qualité du programme CPS actuel.

**Peer Reviewed**
